# Associations between ecological diversity and rodent plague circulation in Yunnan Province, China, 1983–2020: A data-informed modelling study

**DOI:** 10.1371/journal.pntd.0011317

**Published:** 2023-06-22

**Authors:** Ruiyun Li, Chao Su, Zhe Lou, Zhizhong Song, Ennian Pu, Yuqiong Li, Zihou Gao

**Affiliations:** 1 School of Public Health, Nanjing Medical University, Nanjing, Jiangsu, China; 2 Yunnan Institute of Endemic Diseases Control and Prevention, Dali, Yunnan, China; 3 Yunnan Center for Disease Control and Prevention, Kunming, Yunnan, China; University of Florida, UNITED STATES

## Abstract

**Background:**

Following its resurgence in 1982, rodent plague has been linked to a wide range of circulation risks in Yunnan Province. The most serious public health concern associated with effective plague control is determining how various ecological variables influence the differential risk of transmission.

**Methods:**

We investigated the population dynamics of the hosts and vectors using large-scale epidemiological surveillance data. In a seasonal eco-epidemiological model, we evaluated the impact of ecological conditions on the vectored flea index (VFI) to determine the rate of plague transmission.

**Results:**

The findings revealed a changing species composition in natural foci over time. Additionally, shifting distributional ranges of species by elevation may be vital in modulating the VFI. The model estimates indicate that the dynamic VFI contributes to spatiotemporal variance in transmission.

**Conclusions:**

The VFI could be a critical ecological indicator, allowing for real-time tracking and prompt intervention in the circulation of rodent plague. Understanding eco-epidemiological diversity can provide essential insights into effective responses to future plague resurgence.

## Author summary

Plague have resurged among rodents in Yunnan Province since 1982, and intermittently occurs among humans. Yet the local risk of rodent plague dynamics has been highly diverse across ecological contexts. In natural foci, suggestions for this ecological diversity include high species richness and cyclic transmission between hosts and vectors. Here, we developed an eco-epidemiological framework to position the epidemiological dynamics of rodent plague in local ecological contexts. Our results suggest that diverse ecological conditions may have shaped the local risk of plague transmission in Yunnan Province. Typically, incremental of elevation has been associated with the tradeoff between the increased diversity and averted fraction of main host and vector species. More importantly, vectored flea index could be a critical ecological indicator for plague management.

## Background

The rodent plague resurged in Yunnan Province in 1982, following a 26-year absence. High species richness and cyclic transmission between hosts and vectors offer much of the ecological diversity that may be essential for the preservation of natural foci [1,2]. Ultimately, plague occurs naturally among rodents, and intermittently among humans. Manipulating the possible resurgence, spatial expansion, and spillover risk motivates us to position the epidemiological dynamics of rodent plague in local ecological contexts.

Similar to other natural-focal diseases [3], understanding how ecological conditions are likely to impact the course of plague dynamics is a primary concern for plague research. Given the diverse ecological landscapes regarding climate conditions, vegetation cover, and species distribution in Yunnan [4,5,6,7], rodent plague is primarily induced locally with its own epidemic trajectories [8,9]. However, this ecological diversity complicates the characterisation of the plague dynamic process and transmission potential arising from the local ecological system. Investigating the mechanistic dynamics of plague with techniques that combine ecological and epidemiological insights are critical for an effective response to plague circulation and its potential resurgence.

Predicting vectored disease dynamics under changing ecological conditions is challenging, but not impossible [10,11]. We developed a climate-epidemic model [12] that allowed us to include climatic impacts on vector populations in disease dynamics. Model simulations revealed that spatiotemporal disease dynamics vary in response to climate-driven changes in local vector dynamics. This enables the simultaneous inference of vector responses to the natural environment as well as their contribution to disease dynamics. This work expands on such a predictive model by applying the fundamental eco-epidemiological linkage to rodent plague dynamics. More importantly, epidemiological monitoring in Yunnan has grown at an unprecedented rate in recent decades (S1 Fig). These comprehensive records allow for further refinement of the predictive model to elucidate the ecological dependence of plague dynamics.

Using these records, we developed an eco-epidemiological framework to contextualise rodent plague dynamics in local ecological systems. This framework aids in assessing the dynamic risk of transmission among rodents throughout the changing ecological system. In particular, we adjusted the plague ecological landscape by incorporating long-term host and vector surveillance with climate and elevation data in a generalised additive model (GAM). Estimates of the vectored flea index (VFI) were then leveraged as a proxy for the rate of transmission in a susceptible–infected–recovered (SIR) model to integrate plague ecology into its epidemiological dynamics.

## Methods

### Ethics statement

The procedures and protocols for specimen collection and processing in this study were reviewed and approved by the Medical Ethics Committee of the Yunnan Institute of Endemic Disease Control and Prevention (approval number: No.2 in 2023).

### Surveillance data of rodents and fleas

With the geographic dissemination of the plague, systematic surveillance has grown over time (S1 Fig). We collected data from 1983 to 2020 from surveillance reports, including the date and county of the surveillance, the number of cages or traps, the species-stratified number of rodents captured and those carrying fleas, rodent habitats, and the species-stratified number of fleas. Monthly rodent and flea records per county were compiled for epidemiological and modelling analyses.

### Rodent plague cases

Case-level records of rodent plague transmission from 1983 to 2020 were collected from statistics and reports from the Yunnan Institute of Endemic Disease Control and Prevention. The associated information for each case includes the date, village, rodent species, and number of rodents carrying *Yersinia pestis*. The number of rodent plague transmission per month per county was aggregated for mathematical modelling analysis.

### Climatic and ecological data

We collected daily mean temperature and precipitation from the China Meteorological Data and Service Centre for the period 1983–2020 [13]. The obtained climatic data was converted into monthly mean temperature and the number of precipitating days (number of days with precipitation over 1 mm/d) [14]. Furthermore, we collected elevation data from Geospatial Data Cloud [15]. Using the data, we calculated the average elevation for each county in ArcGIS. Climatic and ecological datasets were used for statistical analyses.

### Epidemiological analyses

The prevalence of epidemic sites characterises the spatiotemporal diffusion of rodent plague. We used the National Standard [16] to determine the annual number of epidemic sites across counties in 1983–2020.

Subsequently, we considered the variation in species composition throughout the study period. We first investigated the three primary species that accounted for the majority of the population. We then attempted to relate these population dynamics to rodent density and the flea index. We defined the overall rodent density (*D*) as *D* = *R*^*T*^/*C*×100% and the total flea index (*V*^*T*^) as *V*^*T*^ = *F*^*T*^/*R*^*T*^, where *C* is the total number of cages or traps deployed, and *R*^*T*^ and *F*^*T*^ are the numbers of all rodents and fleas, respectively. Similarly, we calculated the density of rodent species *m* as *D*^*m*^ = *R*^*m*^/*C*×100% and the index of flea species *n* as *V*^*n*^ = *F*^*n*^/*R*^*n*^. We defined the VFI as *V* = *F*^*v*^/*R*^*v*^ where *v* denotes the major vectors. The main plague vectors considered in this study were *Xenopsylla cheopis* in domestic rodent foci and *Neopsylla specialis specialis* and *Frontopsylla spadix spadix* in wild rodent foci [17].

To demonstrate the importance of elevation on the VFI, we examined the spatial distribution of the community structure along elevational gradients. The Shannon-Wiener diversity index was calculated as *H*_*i*_ = −∑_*s*_ *p*_*s*,*i*_ *ln p*_*s*,*i*_ where *p*_*s*,*i*_ is the proportion of species *s* in a population in county *i*. We also quantified the fraction of major vectors and hosts in each county using $f_{i}=p_{s,i}^{\prime }/\sum_{s}p_{s,i}$, where $p_{s,i}^{\prime }$ is the proportion of main vector or host.

### Eco-epidemiological coupling model

Depending on the underlying ecological conditions, rodent plague has varying transmission potential. We developed an eco-epidemiological coupling model that synthesises ecological constraints using the epidemiological dynamics of the plague. We restricted our analysis of the plague ecological system to interactions between hosts, vectors, and the natural environment. To do this, we incorporated the long-term flea and rodent surveillance data, as well as climate and elevation data, into a GAM:

$$V_{t}=a_{t,i}+b(E_{i})+c(T_{t-1,i})+d(P_{t-1,i})+e(V_{t-1,i})+\varepsilon_{t,i}$$ (1)

where *V*_*t*_ is the VFI, defined as the number of plague vectors per rodent (see “Epidemiological analyses”) in month *t* in county *i*. The spatial component *b*(*E*_*i*_) is the average elevation in county *i*. Considering the reproductive period of fleas (3–6 weeks) [18], a 1-month lag between the flea index and local climate conditions is incorporated. Thus, *c*(*T*_*t*−1,*i*_) and *d*(*P*_*t*−1,*i*_) denote the monthly mean temperature and number of rainy days in the last month, respectively. The VFI in the previous month, which takes biological serial dependence into account, is denoted as *e*(*V*_*t*−1,*i*_). The parameters *a*_*t*,*i*_ and *ε*_*t*,*i*_ represent the overall intercept and model error, respectively. We calibrated the GAM using monthly VFI, climate, and elevation data from 34 counties for 1983–2020. The derived empirical associations were then interpolated to provide monthly estimates of the VFI, which were then used as a proxy for transmission rate among rodents in the mathematical model (see below).

The assumption that fluctuation in the VFI over time is proportional to the seasonal rate of plague dynamics in the rodent community illustrates the coupling nature of our eco-epidemiological framework. Similarly, the variation in the VFI caused by the natural environment is proposed as a proxy for plague transmission rate in an epidemiological SIR model described by the following equations:

$$\frac{dS}{dt}=-\frac{\beta^{\prime }(t)\hat{V}SI}{N}$$ (2)

$$\frac{dI}{dt}=\frac{\beta^{\prime }(t)\hat{V}SI}{N}-\gamma I$$ (3)

$$\frac{dR}{dt}=\gamma gI$$ (4)

where *S*, *I*, and *R* are the number of susceptible, infected, and recovered rodents, respectively. The rodent population size is calculated as *N* = *S*+*I*+*R*. $\hat{V}$ represents the monthly VFI estimated using the GAM. Vector efficacy, *β*′(*t*), is the time-varying scaling factor linking the estimated VFI to the transmission rate among rodents. Due to the coupling nature, the transmission rate among rodents can be calculated as a function of *β*′(*t*) and $\hat{V}$ through $\beta (t)=\beta^{\prime }(t)\hat{V}$. Parameter 1/*γ* denotes the average infectious period. Though inconclusive, challenge experiment findings showed that infectious period can vary greatly depending on bacterial concentrations [19]. To address the large variation of 1/*γ*, we considered the parameter to be a random constant from a 7–26 days uniform distribution. Accordingly, the effective reproductive ratio was calculated as *R*_*e*_ = *R*_0_*S*(*t*)/*N*, where $R_{0}=\beta^{\prime }(t)\hat{V}/\gamma$. The recovery rate, denoted as *g*, was set as 0.02 in the study [20] due to the high risk of mortality after infection.

We calibrated the SIR model to the observed number of rodent plagues in outbreak years in three counties (Ruili, Yingjiang, and Longchuan) linked with the earliest records of circulation and hence a long-term burden of rodent plague [21]. Assuming homogeneous susceptibility in the rodent population, we reinitialised the model 500 times with varying *γ* values at the beginning of each outbreak year to simulate rodent plague incidence and assess vector efficacy. The median estimates and 95% confidence intervals of plague incidence from all simulations using varying *γ* values are presented. Median estimates of *β*′(*t*) for each of the three counties were also obtained. We projected rodent dynamics in other counties over time using the median estimates of these time-varying *β*′(*t*). The projections describe the baseline transmission potential in the local context, which is determined by vector efficacy and the VFI. We estimated the yearly average and peak magnitudes of *R*_*e*_ to assess the county-specific transmission potential. We then used linear regression to identify the association between the VFI and the annual average and peak magnitude of *R*_*e*_.

## Results

**Fig 1:**
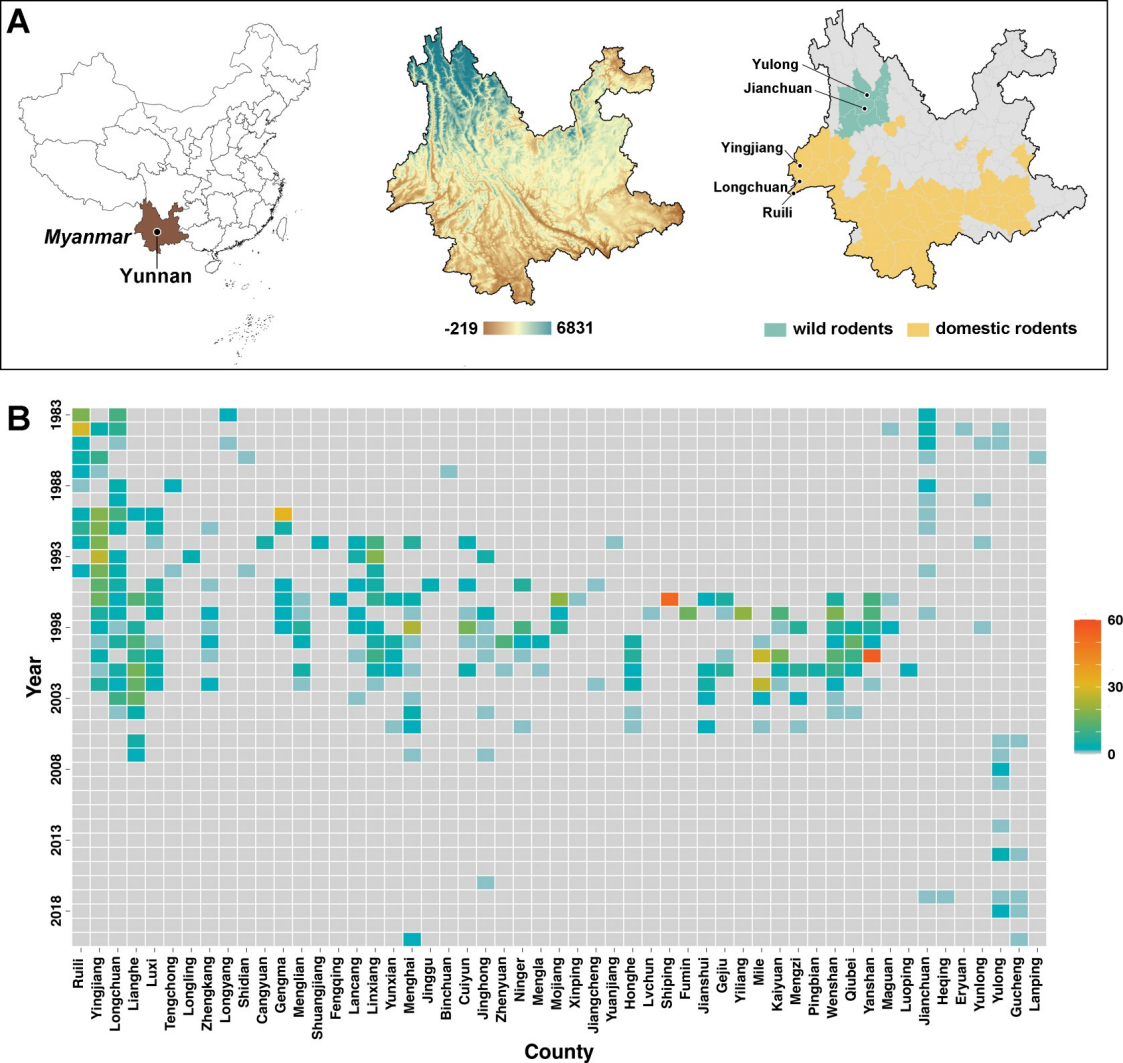
Plague natural foci and epidemic spots in Yunnan. (A) The geographic location and elevation, as well as the plague foci of domestic and wild rodents, are indicated to establish the analysis in the context of Yunnan. Natural foci of domestic (yellow) and wild (green) rodents are distinguished. Counties associated with the earliest records of plague circulation in domestic foci, i.e., Ruili, Yingjiang, and Longchuan, are marked. Counties with a high prevalence of plague circulation in wild rodent foci, i.e., Jinchuan and Yulong, are also labelled. (B) The prevalence of epidemic spots. The annual number of epidemic spots across counties are visualized by colour. Counties in the domestic rodent foci are ordered according to the west-to-east longitudinal gradient. Base map is available from: https://www.tianditu.gov.cn/and https://yunnan.tianditu.gov.cn/.

Surveillance of epidemic hotspots reveals that the varied patterns of plague circulation are determined by natural foci (Fig 1). The majority of epidemic sites within domestic rodent foci were distributed in low-latitude and low-elevation areas, encompassing a wide longitudinal range of counties (Figs 1A and S2). Notably, early records of circulation were found for counties along the China-Myanmar border, namely, Ruili, Yingjiang, and Longchuan (Fig 1B) and a higher proportion of rodents infected with *Yersinia pestis* than other counties (see S3 Fig). This implies that the early stage plague dynamics could potentially be determined in these counties. Beginning in these border counties, plague dynamics in domestic foci appear to be linked to a clear west-to-east spatial dissemination, as well as a highly active period from 1990 to 2005 (Fig 1B). In contrast, epidemic sites within wild rodent foci were concentrated in high-latitude and high-elevation areas (Figs 1A and S2). Despite the lack of evident spatial diffusion, plague epidemic dynamics within wild foci were characterised by a primary cluster centred around Jianchuan in 1983–1990 and an emerging cluster surrounding Yulong since 2006 (Fig 1B).

**Fig 2:**
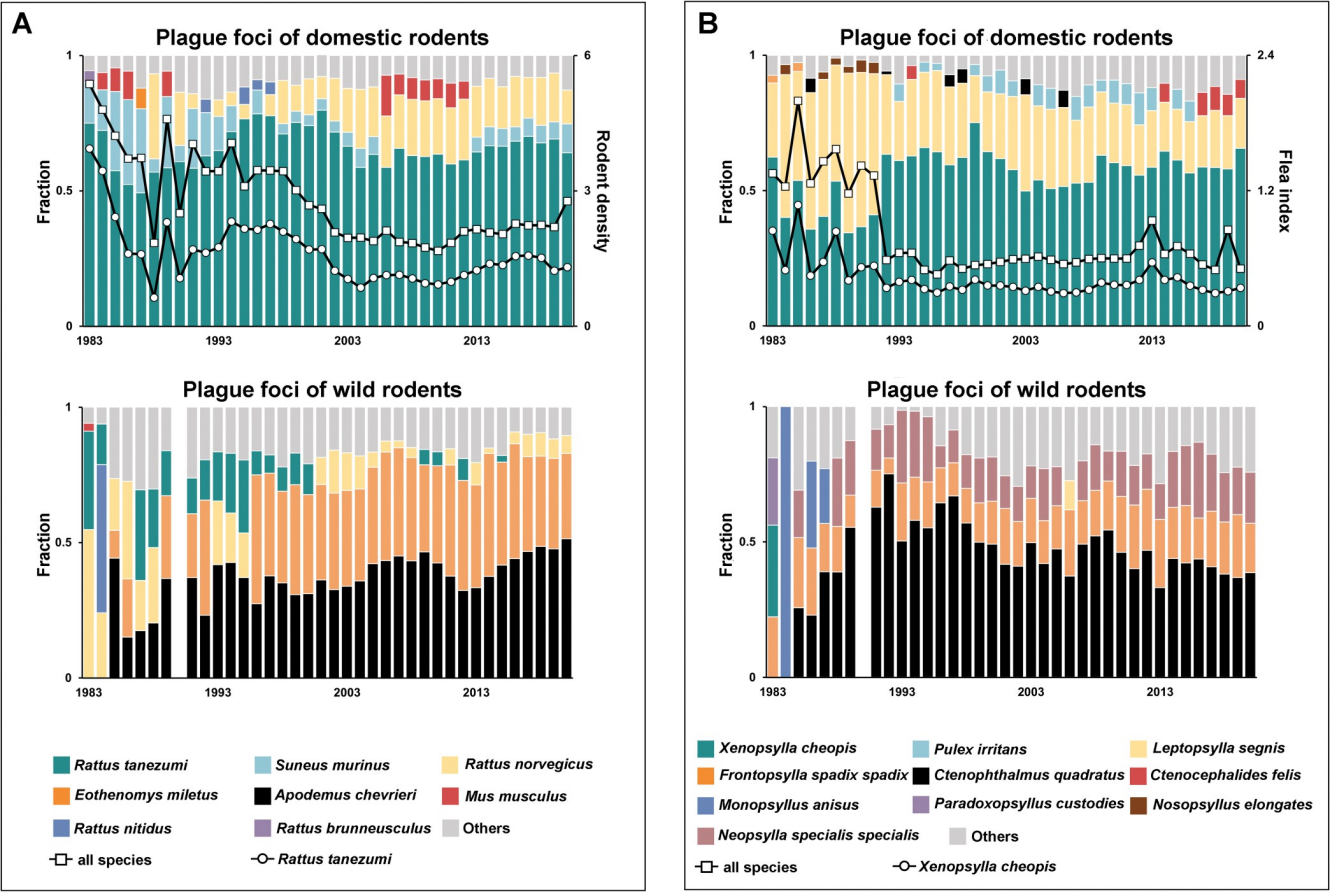
Population dynamics of rodents and fleas in 1983–2020. (A) The three primary rodent species found in domestic and wild rodent foci over the study period are marked by colour. (B) The annual flea species composition in two foci.

The rodent species composition varied over the study period (Fig 2A). Notably, we discovered a fraction trade-off between *Rattus tanezumi* and secondary species in domestic foci. Given their similar spatial distributions, it is highly possible that the trade-off between *Rattus tanezumi* and *Suneus murinus* prior to 1997 was the result of species competition (S4 Fig). Interestingly, the secondary species changed from *Suneus murinus* to *Rattus norvegicus* in 1997. This shift could be attributed to the west-to-east extension of monitoring, which corresponds roughly to the geographical diffusion of the epidemic (S1 Fig), rather than a competition between species. Accordingly, the rise in *Rattus norvegicus* may partially contribute to a gradual decrease in the fraction and hence the density of *Rattus tanezumi*. The proportion of *Eothenomys miletus* in wild rodent foci has been increasing since 1996 (Fig 2A). This increase characterises the species composition of outdoor habitats (S5 Fig), which account for approximately 86% of all rodents.

Similar to the rodent population, the annual composition of flea species was dynamic (Fig 2B). We discovered a marked change in the proportion of *Xenopsylla cheopis* and *Leptopsylla segnis* in domestic rodent foci around 1992, suggesting that that have competitive relationships since they parasitise the same rodent species. The initial population decline of *Xenopsylla cheopis* corresponded to a rapid decrease in the flea index during 1983–1991. However, the population has grown to an average of approximately 60% of all species since 1992, reducing the share of *Leptopsylla segnis* to approximately 26%. We found a similar interaction of population dynamics between *Xenopsylla cheopis* and *Leptopsylla segnis* isolated from *Rattus tanezumi* when we focused on the dominant rodent species (S6 Fig). In comparison, the flea composition in wild rodent foci was highly dependent on host species (Figs 2B and S7). We discovered that the flea population on *Eothenomys miletus* varied minimally, with *Ctenophthalmus quadratus* typically accounting for more than 85%. In contrast, the annual proportion of *Ctenophthalmus quadratus* isolated from *Apodemus chevrieri* remained under 20%, peaking at approximately 48% in 1995. *Neopsylla specialis* and *Frontopsylla spadix* collectively accounted for 80% of the fleas isolated from *Apodemus chevrieri* over the past decades, except for a ~50% reduction around 1995. The species composition in the early years is likely to have varied because more rodents were collected.

**Fig 3:**
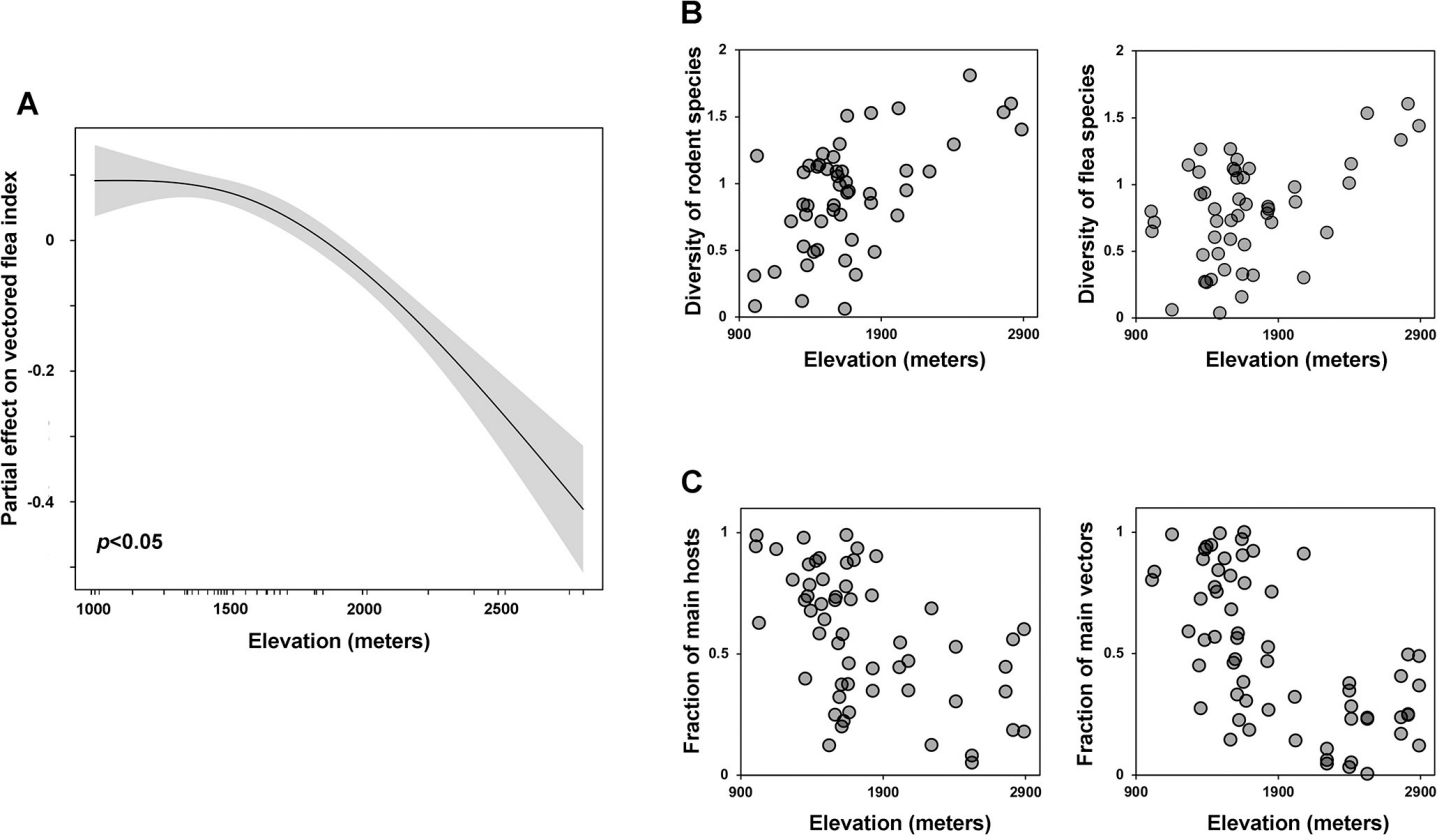
Statistical association between VFI and natural environment. (A) The partial effects of elevation on VFI are quantified using GAM. (B-C) Spatial distribution of community structure of fleas and rodents along elevational gradients. The diversity of flea and rodent species in each county is estimated using the Shannon index. The fraction of main vectors and hosts in the entire flea and rodent population in each county are also estimated. The estimates of diversity and fractions are plotted against country elevations.

Next, we used geographic and meteorological data to characterise the associations between fleas, rodents, and the natural environment. The VFI was substantially correlated with county elevations (Fig 3A). It should be noted that incremental elevation has a waning influence on the VFI. We quantified the geographical distribution of rodent and flea populations along elevational gradients to help explain this association. We accomplished this by evaluating species diversity using Shannon’s index. We discovered that diversity increased in tandem with elevation (Fig 3B). This corresponds to a diminishing fraction of the primary hosts and vectors as elevation increases (Fig 3C). Therefore, elevation can have a strong impact on the distributional limitations of species and, consequentially, drive the VFI. Low-elevation regions tended to have lower diversity and a higher share of major hosts and vectors, resulting in the most pronounced impact of elevation on the VFI. Additionally, we discovered a growing association between the vectored flea index and local climatic conditions (S8 Fig). The rising temperature and frequency of rainy days may generate a humid and warm ambient environment, increasing the chances of flea survival and transmission, and hence, increased regulation of the flea index. However, the negligible climate-index association suggests that elevation determines local climatic conditions and therefore the distributional boundaries of fleas and rodents, thereby hindering the direct influence of climate on the VFI.

**Fig 4:**
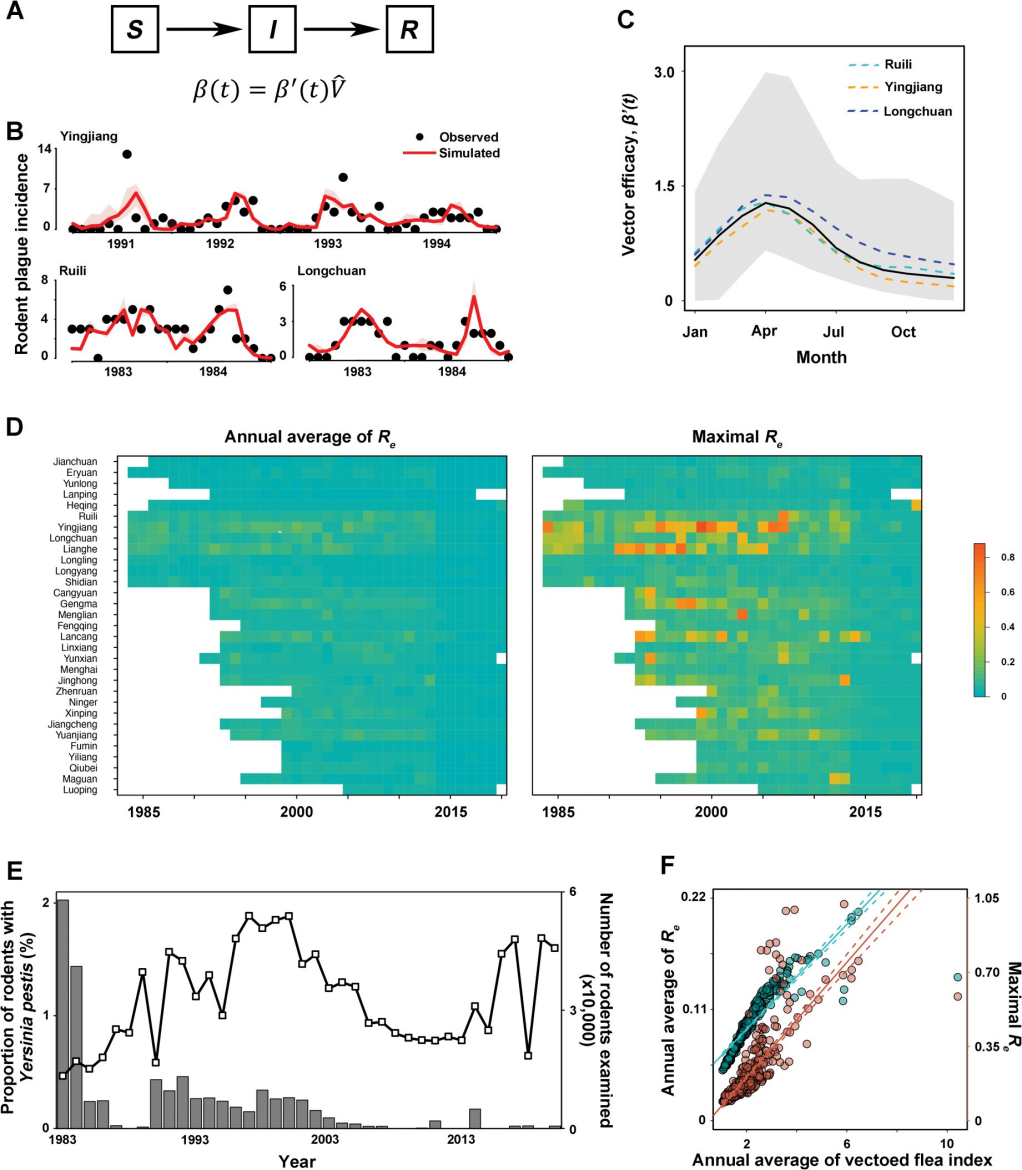
Estimates of transmission potential in rodent community. (A) Coupling of the estimates of VFI with epidemic compartmental SIR model. Time-varying transmission rate *β*′(*t*) is dependent on VFI, $\hat{V}$, and vector efficacy, *β*′(*t*). (B) Observed and simulated number of rodent plagues in outbreak years in three representative counties. (C) Estimates of vector efficacy. Dash lines represent the median estimates of the efficacy across varying values of removal (recover or death) rate, 1/*γ*, for three counties. Solid line and shade area are the median estimates and 95% CI among these counties, respectively. (D) Median estimates of the annual average and peaking magnitude of effective reproductive ratio (*R*_*e*_) over years and counties. (E) Proportion of rodents with Yersinia pestis (bars) among those examined (lines) over years. (F) Associations between the annual average of VFI and the mean and peaking magnitude of *R*_*e*_ over a year.

The vector is the primary regulator in the fundamental flea-rodent cyclic transmission of rodent plague in empirical settings. Therefore, we focused on the inference of vectored plague transmission. Accordingly, we propose that the VFI and vector efficacy work together to determine seasonal variation in the transmission rate in an SIR model (Fig 4A), and hence epidemic trajectories. The fitted incidence curve captures the dynamics of rodent plagues in counties with the earliest records of circulation (Fig 4B). Furthermore, seasonal fluctuation in vector effectiveness was highly consistent throughout the three counties, with a peak around April (Fig 4C). Given this consistency, we projected epidemic trajectories in other counties using the median estimates of these efficacies. As a result, we expected that efficacy will vary minimally between counties, and we established the baseline estimates of transmission. We estimated that the annual average and peak magnitudes of *R*_*e*_<1 (Fig 4D). This finding implied that the foci have a low overall transmission potential. To support the baseline estimates, we considered the share of rodents infected with *Yersinia pestis*, which has indicated a marginal fraction of infected rodents (<0.5%) since 1985 (Fig 4E). Furthermore, we examined the contribution of the VFI to the variation in *R*_*e*_. Fig 4F demonstrates that the transmission potential in the rodent population was significantly correlated with the VFI (p<0.05). Collectively, these results suggest that the VFI, which is influenced by the local natural environment, may influence the rates and hence trajectories of plague epidemics in rodents.

## Discussion

The incorporation of systematic surveillance and a predictive framework facilitates the accurate characterisation of plague circulation. By situating our analysis in the context of Yunnan, we provide a striking illustration of the critical need to link plague epidemiological dynamics with local ecological conditions. We propose a highly dynamic ecological system as the foundation for plague circulation. It is worth noting that species competition and elevation may be critical influences shaping the evolving ecological environment. During the intervention-free period, species competition may contribute to population shrinkage, and thus, a decrease in *Rattus tanezumi* density. Furthermore, elevation may disrupt host–vector associations, affecting both local climatic conditions and species distributional ranges. Species may be responsive to climate change by shifting their range [12]. The colder and drier climate at higher elevations may dramatically reduce the percentage of primary vector and host species, resulting in a diminished elevation impact on the VFI. These findings are consistent with recent research [5,7] indicating the dominance of elevation in the community structure of hosts and vectors known to be responsible for plague circulation.

Plague research has prioritised accurate evaluation of plague transmission potential for effective plague management. Our findings indicate that the county-level transmission potential was generally low. This relatively low transmission potential may be largely attributable to the heterogeneous geographical distribution of rodents, fleas, and *Yersinia pestis* [22,23]. These heterogeneities, which are promoted by a heterogeneous natural environment, may have shaped the granular difference in plague recurrence risk, which is thus therefore within each county. Beyond the overall estimates, the potential for plague transmission varies by county and year. This transmission diversity appeared to be driven by the local VFI, which is controlled by natural environmental changes with elevation. Therefore, we highlighted the potential of VFI as a key ecological indicator for real-time risk assessment of rodent plague dynamics. Our focus on key plague vectors was consistent with the fact that various flea species may have varying capacities to generate bacterial emboli and hence contribute to plague circulation [20]. Alternatively, the conventional total flea index, which includes non-vector species, may not accurately characterise the risk of plague transmission. Continuous surveillance of host and vector populations will provide valuable information for assessing rodent plague circulation and customising local public health interventions.

More importantly, we proposed a seasonality of vector efficacy that has conventionally been difficult to observe. The efficacy, which peaked around April, suggested that fleas have an increased capacity to transmit pathogens among rodents in the early spring. In relation to the peak season of plague circulation (June–August) [1], this seasonal-varying efficacy may convey an advanced 1–2-month plague outbreak warning. Initialising interventions around April might thus result in an overall decrease in rodent plague in subsequent months, allowing the control of plague circulation in natural foci to be maintained.

## Conclusions

By synergising insights from ecology and epidemiology, our study contributes to shifting the focus of plague research. We demonstrated that hosts, vectors, and the natural environment determine the various ecological variables that underpin rodent plague transmission. Large-scale epidemiological surveillance reveals a variety of ecological conditions, aiding in the accurate characterisation of dynamic rodent circulation in natural foci. The general inference framework and ecological indicators proposed here serve as tools that enable early warning and prompt and effective responses to plague circulation.

## Supporting information

S1 Text The supplementary file of this study includes the following information: Supplementary Methods, Supplementary Results, Supplementary Discussion, Supplementary References. (DOCX)

S1 Fig Spatial extension of the epidemic and surveillance. Counties with records of rodent plague and surveillance sites of rodents and fleas in (A) 1983, (B) 1984–1988, (C) 1989–1995 and (D) 1996–2001. The surveillance covers all the 104 counties since 2001 and thereby is not illustrated. Base map is available from: https://yunnan.tianditu.gov.cn/
(TIF)

S2 Fig Distribution of epidemic spots. The distribution of epidemic spots along (A) longitudinal, (B) latitudinal and (C) elevational gradients and (D) over years is distinguished by plague natural foci.
(TIF)

S3 Fig The proportion of rodents with *Yersinia pestis* during 1983–2020. Counties with the earliest records of rodent plague circulation are marked. Base map is available from: https://yunnan.tianditu.gov.cn/
(TIF)

S4 Fig Spatial distribution of rodent species in domestic rodent foci. The distribution of *Rattus tanezumi*, *Suneus murinus*, *Rattus norvegicus* at the intersection of longitudinal and elevational gradients are presented. Circle size characterizes the total number of rodents captured over the study period.
(TIF)

S5 Fig Rodent species composition among the indoor and outdoor habitats in wild rodent foci. Same with Fig 2A but distinguished by the (A) outdoor and (B) indoor habitats. Overall, 85% of records are of known habitats. Habitats of all records in 1983 are unavailable.
(TIF)

S6 Fig Flea species composition on *Rattus tanezumi* in domestic rodent foci. Same with Fig 2B but focus on the flea species isolated from the main host in domestic foci (*Rattus tanezumi*).
(TIF)

S7 Fig Flea species composition on *Eothenomys miletus* and *Apodemus chevrieri* in wild rodent foci. Same with Fig 2B but focus on the flea species isolated from main hosts in wild foci, i.e. (A) *Eothenomys miletus* and (B) *Apodemus chevrieri*.
(TIF)

S8 Fig Statistical association between VFI and natural environment. (A) The partial effects of elevation and meteorological conditions i.e. (B) monthly mean temperature and (C) precipitating days in the previous month on VFI are quantified using GAM.
(TIF)

S9 Fig Estimates of VFI in outbreak years in three representative counties. i.e. (A) Yingjiang, (B) Ruili and (C) Longchuan.
(TIF)

S10 Fig Interdependence of rodent and flea species. The dominant, secondary and other species of rodents and fleas in domestic (yellow) and wild (green) rodent foci are shown. The size of the arc of sectors is the fraction of species in the population.
(TIF)

S11 Fig Estimates of rodent plagues in outbreak years in three representative counties by alternative model. Estimates of rodent plague in (A) Yingjiang, (B) Ruili and (C) Longchuan by the main model and alternative model are presented by red and green curves, respectively. Observed number of rodent plague are shown by points.
(TIF)

S1 Table Species distribution in the natural foci of domestic rodents. The species-stratified number and proportion of flea-infested rodents, as well as the corresponding number and share of different flea species.
(XLSX)

S2 Table Species distribution in the natural foci of wild rodents. The species-stratified number and proportion of flea-infested rodents, as well as corresponding numbers and shares of different flea species.
(XLSX)

S3 Table Statistical model performance in fitting VFI. The performance of GAM in selected (i.e., the 1-month lag model) and all counties (i.e., the full model) in two foci is quantified by the generalized cross-validation criterion (GCV), proportion of deviation explained by model and R^2^.
(DOCX)
