# Supplementary material for: Associations between ecological diversity and rodent plague circulation in Yunnan Province, China, 1983–2020: A data-informed modelling study
Source: PLoS Negl Trop Dis. 2023 Jun 22;17(6):e0011317. doi: 10.1371/journal.pntd.0011317 (PMC10287002; doi:10.1371/journal.pntd.0011317)
Supplement: S1 Text — (DOCX) [file pntd.0011317.s001.docx]

**S1 Text**

**Supplement to:** Li R, Su C, Lou Z, et al. Associations between ecological diversity and rodent plague circulation in Yunnan Province, China, 1983–2020: A data-informed modelling study

**1. Supplementary Methods**

**Surveillance of rodents and fleas**

Routine surveillance of rodents and fleas in 1983–2020 was directed by Yunnan Institute of Endemic Diseases Control and Prevention. Across years the systematic surveillance has been expanded with the spatial dissemination of plague. In brief, the surveillance is initialized from 34 counties along the national border in 1983, and extended to an additional of 17 counties during 1984–1988. The effort of surveillance has then been rapidly enhanced due primarily to the incremental budget since 1988, covering the entire province (i.e. 104 counties) since 2001 (see S1 Fig).

Rodent surveillance approach differs between indoor and outdoor habitats. [1,2] Precisely, the indoor surveillance in urban and rural areas was implemented by using cages. A minimum of 5-10 cages are deployed in 10-20 households per site per day. While the outdoor surveillance was conducted by arranging mouse traps. The traps were deployed using the 5-meter wire-clamping method among diverse habitats, including farmland, vegetable garden and woodland. Across habitats surveillance were carried out for three consecutive days, generating a minimum of 300 traps per habitat per month. Cages or traps were deployed in the evening and collected in the morning the next day. The collected rodents were bagged and shipped to laboratories for subsequent analyses.

To characterize population constitution, species identification was firstly implemented. To accomplish this, we designated rodent species by morphological identification method. [3] We then preserved fleas isolated from anesthetized rodents in 70% ethanol. [1,2] Slide specimens of fleas were made by a gradual dehydration using 30%, 50%, 70%, 90% and 95% ethanol, followed by a transparent processing using ethanol and xylene. With specimens, we identified flea species by morphological methods using microscope. To further consider the prevalence of infected rodents, we made bacteriological examination of rodents. We did this by inoculating tissues of the collected rodents on selective sensitive medium for a 3-day observation under 28℃. Suspicious *Yersinia pestis* colonies were re-inoculated on selective sensitive medium for purity test.

**Rodent plague cases**

Case-level records of rodent plague circulation in 1983–2020 were obtained from the statistics and reports of Yunnan Institute of Endemic Disease Control and Prevention. The associated information of each case includes the date, village, rodent species, and the number of rodents with Yersinia pestis. A record where the number of rodents with *Yersinia pestis* is greater than 0 (hereafter “positive record”) corresponds to one circulation. Irrespective of rodent species, positive records for the same day and village are considered as the result of one circulation. The number of rodent plague circulations per month per county were aggregated for mathematical modelling analysis.

**Alternative epidemiological model**

We further investigated how the inclusion of VFI as the proxy for transmission rate improves model inference of plague dynamics. To do this, we developed an alternative model where VFI ($\hat{V}$) is not considered in the transmission rate. Such that transmission rate is determined by vector efficacy and defined as $\beta(t)=\beta^{'}(t)$. Accordingly, the alternative epidemiological SIR model is described by the following equation (5)-(7). Definition of variables and parameters are same with those in the main text.

$\frac{dS}{dt}=-\frac{\beta^{'}(t)SI}{N}$ (5)

$\frac{dI}{dt}=\frac{\beta^{'}(t)SI}{N}-\gamma I$ (6)

$\frac{dR}{dt}=\gamma gI$ (7)

**2. Supplementary Results**

**Quantitative characteristics of hosts and vectors.** Overall, we collect a total of 347,941 rodents carrying fleas, with 54% (187,865) and 46% (160,076) in domestic and wild rodent foci, respectively. We first sought to identify the interplay of rodent and flea species (see S9 Fig and S1 and S2 Tables). Our surveillance results demonstrates that *Rattus tanezumi* (80%, 150,841 out of 187,865) is the dominant rodent species in domestic foci, mainly carrying *Xenopsylla cheopis* and *Leptopsylla segnis* flea species. Of the species in wild rodent foci, *Eothenomys miletus* (42%, 66,875 out of 160,076) and *Apodemus chevrieri* (34%, 54,243 out of 160,076) are dominant rodent species. However, they differ with respect to the parasitic flea species. We show that *Ctenophthalmus quadratus* takes up of 88% of the isolated fleas and thereby being the dominant species of *Eothenomys miletus*; whereas *Neopsylla specialis specialis* and *Frontopsylla spadix spadix*, constituting 41% and 39% of the isolated fleas, dominate the flea species on *Apodemus chevrieri*.

**Sensitivity analysis.** 17 counties in the foci was excluded in the calibration of GAM due to its limited number of records on VFI. Sensitivity analysis was made to validate model calibration by including all counties in two foci, i.e. the full model. Model performance was evaluated and compared using the GCV, proportion of deviations explained and R^2^. The analyses were implemented using the mgcv package in R. Sensitivity analysis suggests minimal impact of excluding counties with biased records on the findings (see S3 Table). Further, we simulated plague dynamics by using alternative epidemiological model where transmission rate is determined by vector efficacy and defined as $\beta(t)=\beta^{'}(t)$. The findings show that the predicted number of plague dynamics using alternative model falls to capture the dynamics of rodent plague (S11 Fig).

**3. Supplementary Discussion**

We acknowledged that there might be a proportion of undocumented rodent plague circulations. We thereby might underestimate the transmission potential in rodent community. Nevertheless, the true transmission potential of rodent plague will closely match our estimates, given the comprehensive records drawn from large-scale surveillance.

Moving forward, it is necessary to consider several promising directions to reach the full potential of our eco-epidemiological predictive framework for natural-focal diseases. It is possible that rodent population dynamics may be prone to other regulators. One additional regulator may be the massive rodent control campaign implemented since 2000. Investigating the impact of such intervention on distributional shift of species and thereby reduction of rodent density may better guide policy decisions on plague management. Additionally, disentangling species-stratified response to natural environment and incorporating flea searching efficacy [4] will help add granularity to future models. It is noted that species composition itself is a spatially-continuous phenomenon, suggesting that considering species composition among adjacent sampling units would be crucial. Furthermore, considering the divergence regarding susceptibility and resistance to pathogens [5] is an essential step towards projecting our framework to various settings. We expect that settings with larger fraction of susceptible hosts and limited resistance against pathogens are likely to encounter circulations of higher risk. Lastly, understanding how the uncertainties may result in deviations from our baseline estimates of plague dynamics warrants further studies. Key uncertainties that remains are the duration of infection among rodents and the vector efficacy in the wild rodent foci.

**4. Supplementary References**

1 The Ministry of Health of the People’s Republic of China. National Plague Monitoring Program. China; 2005 p. 1–15.

2 Yunnan Plague Mornitoring Program. 2008.

3 A field guide to the mammals of China. Edited by Pan J. Chinese Forestry Publishing House, Beijing. 2007.

4 Dean KR, Krauer F, Walløe L, Lingjærde OC, Bramanti B, Stenseth NC, et al. Human ectoparasites and the spread of plague in Europe during the Second Pandemic. *Proc Natl Acad Sci U S A* 2018;**115:**1304–9.

5 Andrianaivoarimanana V, Kreppel K, Elissa N, Duplantier JM, Carniel E, Rajerison M, et al. Understanding the Persistence of Plague Foci in Madagascar. *PLoS Negl Trop Dis* 2013;**7:**1–8.
