# Supplementary figures and images for: Associations between ecological diversity and rodent plague circulation in Yunnan Province, China, 1983–2020: A data-informed modelling study
Source: PLoS Negl Trop Dis. 2023 Jun 22;17(6):e0011317. doi: 10.1371/journal.pntd.0011317 (PMC10287002; doi:10.1371/journal.pntd.0011317)

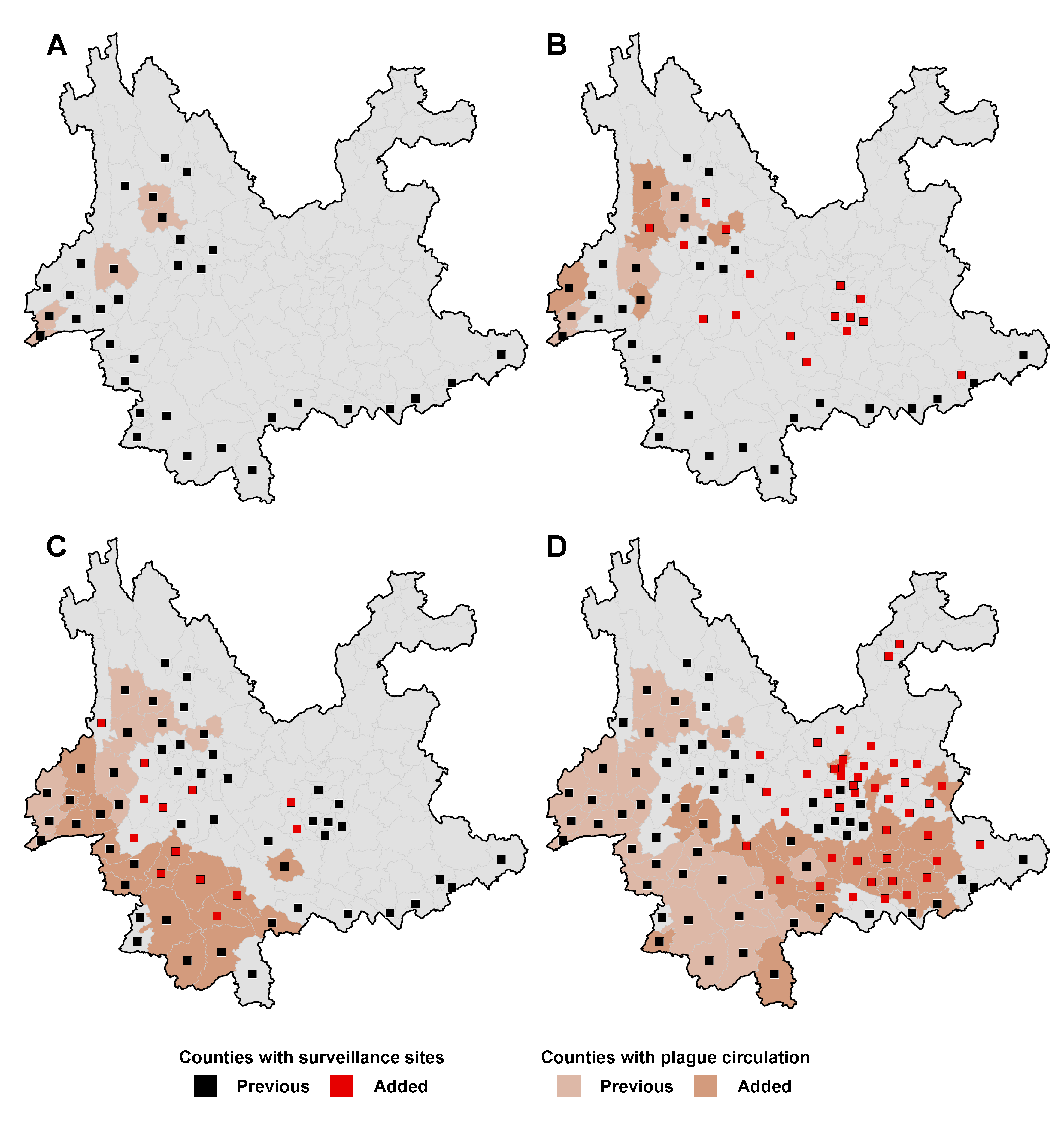

Supplement: S1 Fig — Counties with records of rodent plague and surveillance sites of rodents and fleas in (A) 1983, (B) 1984–1988, (C) 1989–1995 and (D) 1996–2001. The surveillance covers all the 104 counties since 2001 and thereby is not illustrated. Base map is available from: https://yunnan.tianditu.gov.cn/ (TIF) [file pntd.0011317.s002.tif]

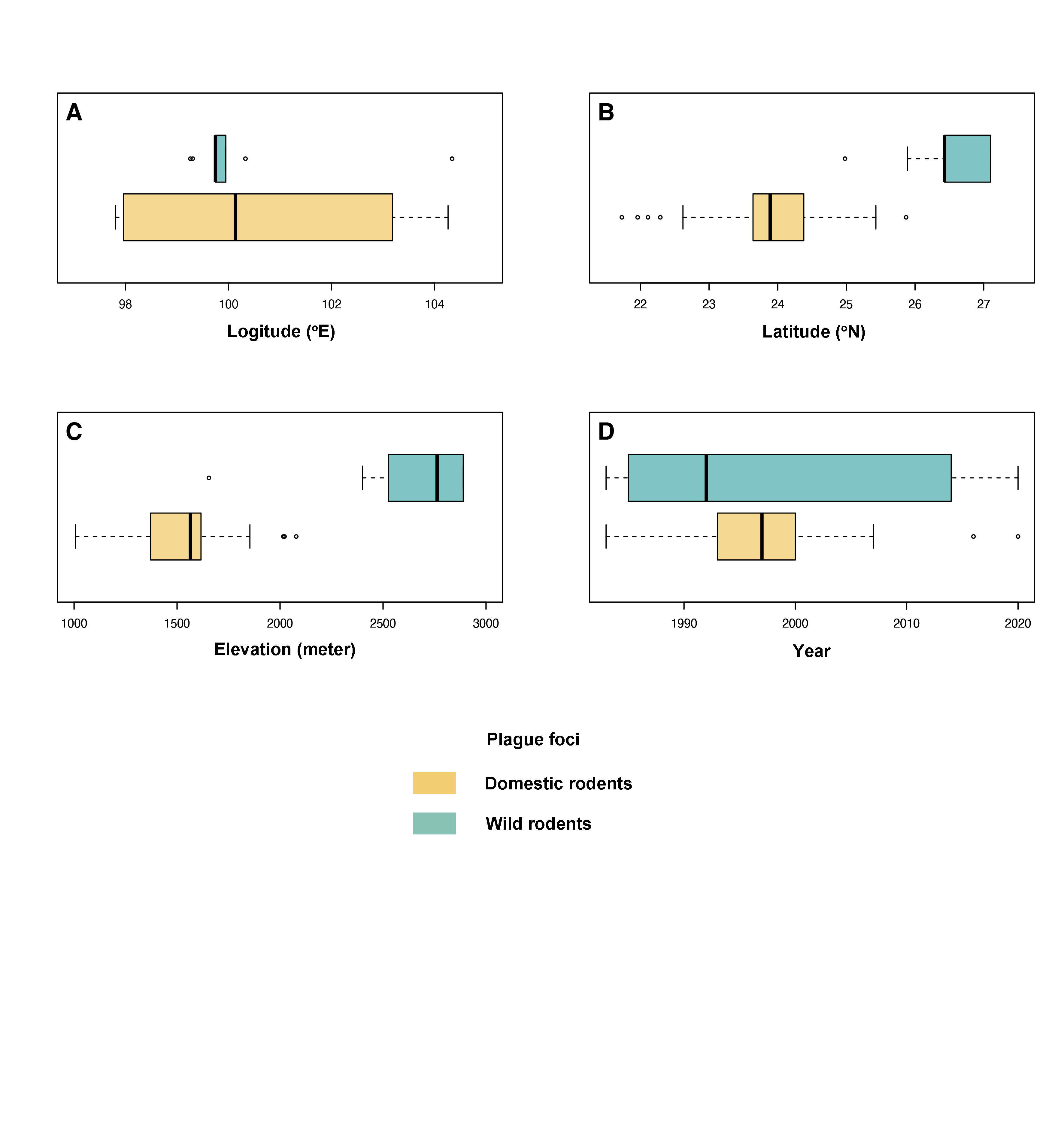

Supplement: S2 Fig — The distribution of epidemic spots along (A) longitudinal, (B) latitudinal and (C) elevational gradients and (D) over years is distinguished by plague natural foci. (TIF) [file pntd.0011317.s003.tif]

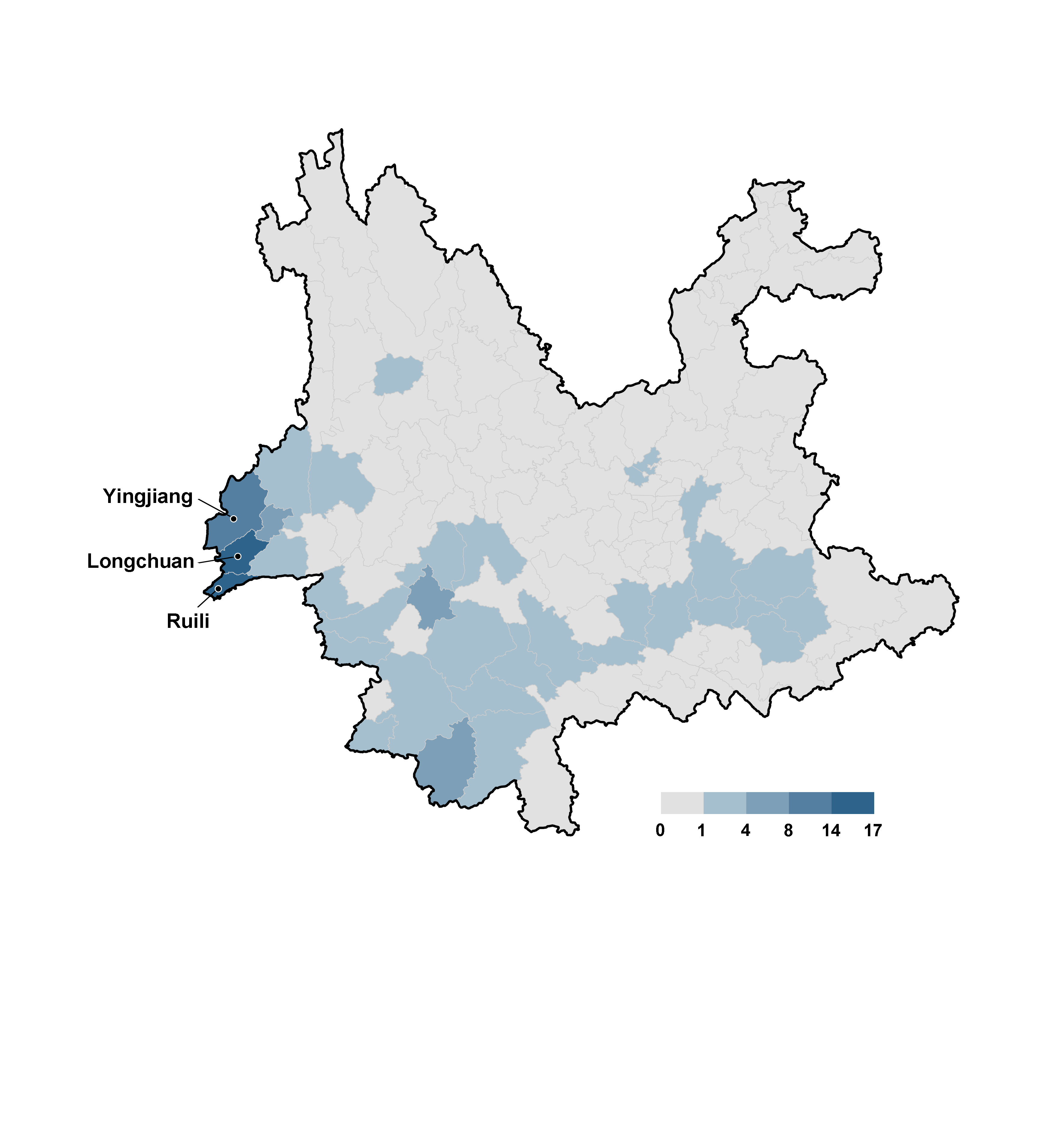

Supplement: S3 Fig — Counties with the earliest records of rodent plague circulation are marked. Base map is available from: https://yunnan.tianditu.gov.cn/ (TIF) [file pntd.0011317.s004.tif]

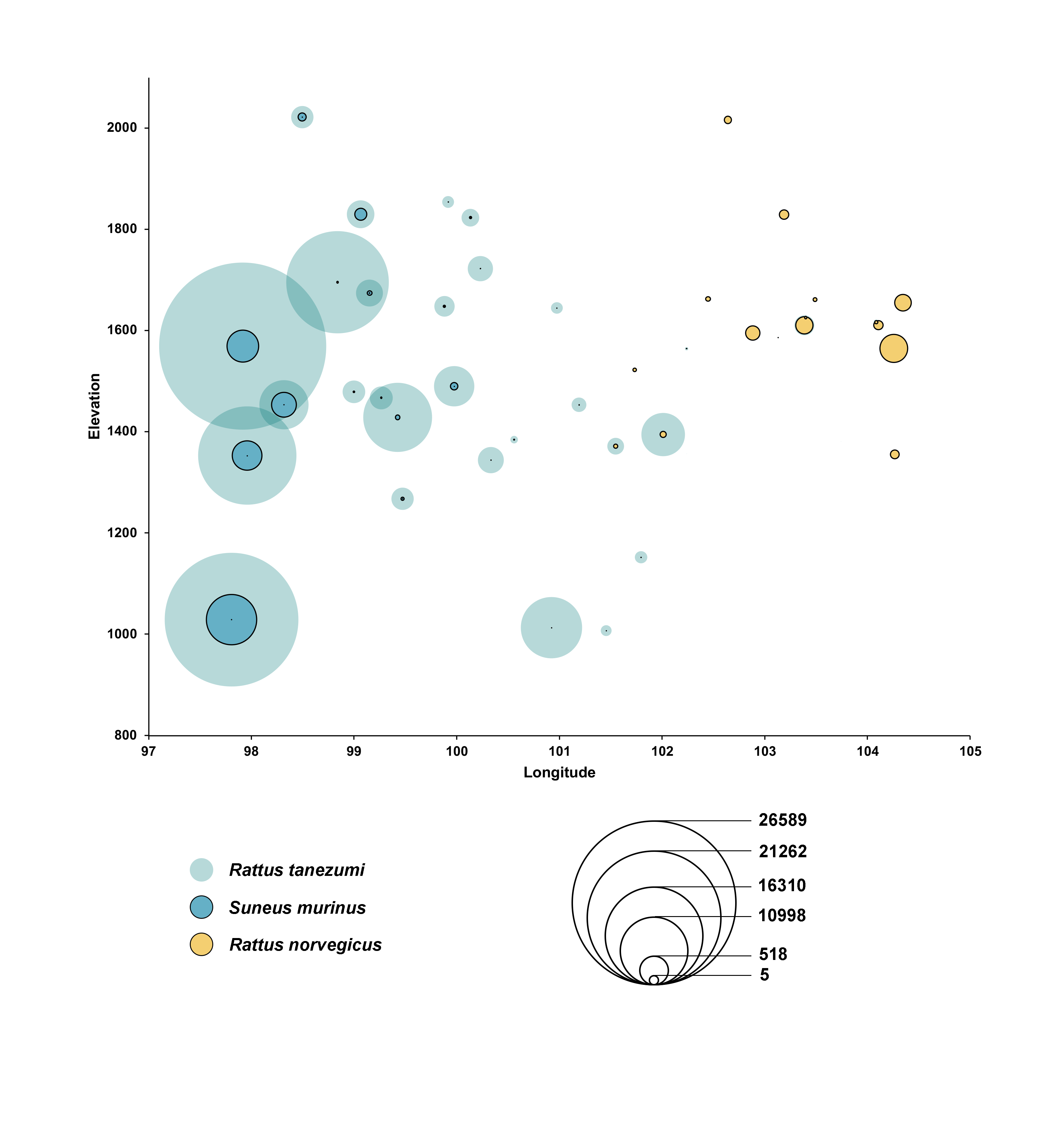

Supplement: S4 Fig — The distribution of Rattus tanezumi, Suneus murinus, Rattus norvegicus at the intersection of longitudinal and elevational gradients are presented. Circle size characterizes the total number of rodents captured over the study period. (TIF) [file pntd.0011317.s005.tif]

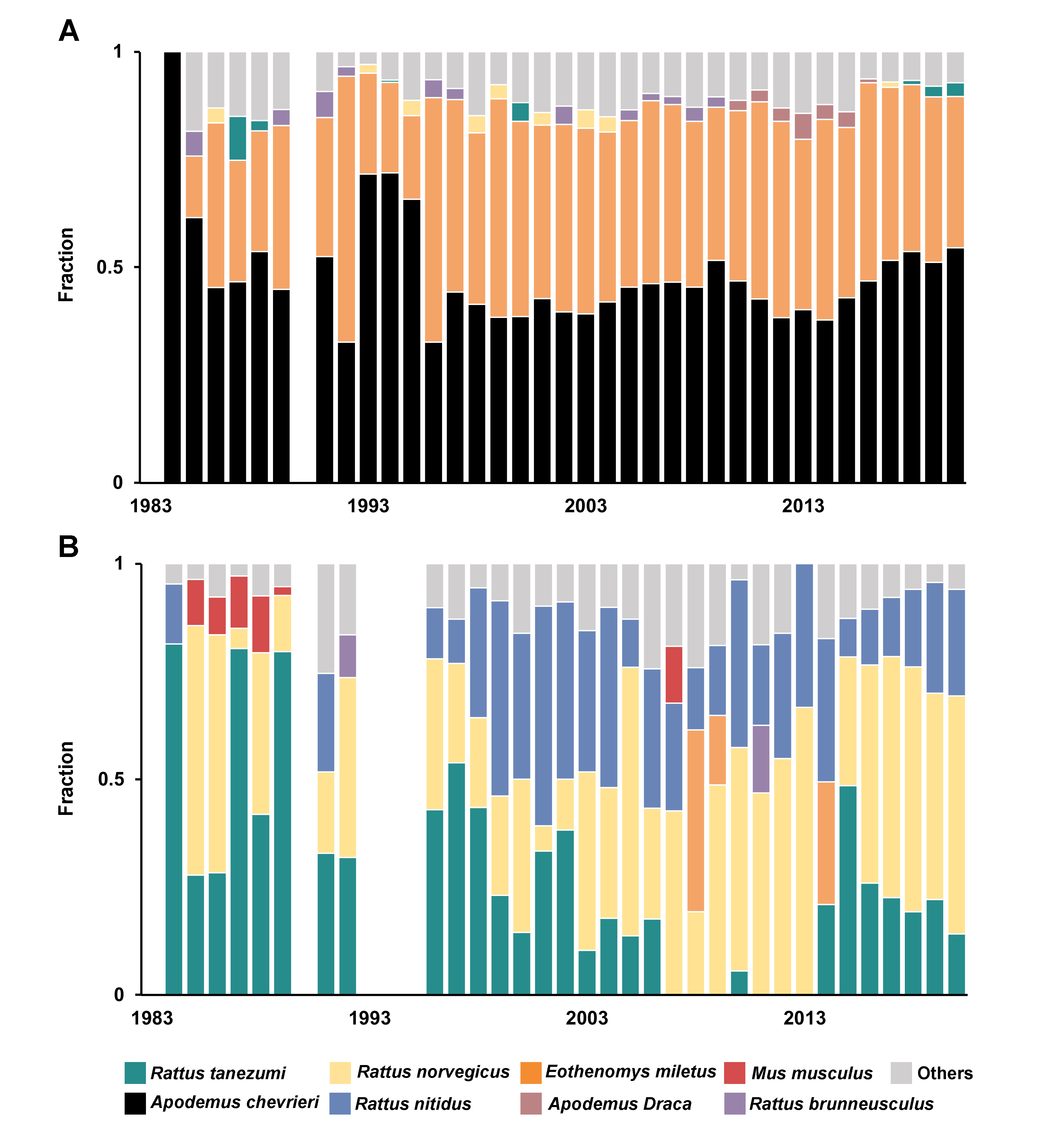

Supplement: S5 Fig — Same with Fig 2A but distinguished by the (A) outdoor and (B) indoor habitats. Overall, 85% of records are of known habitats. Habitats of all records in 1983 are unavailable. (TIF) [file pntd.0011317.s006.tif]

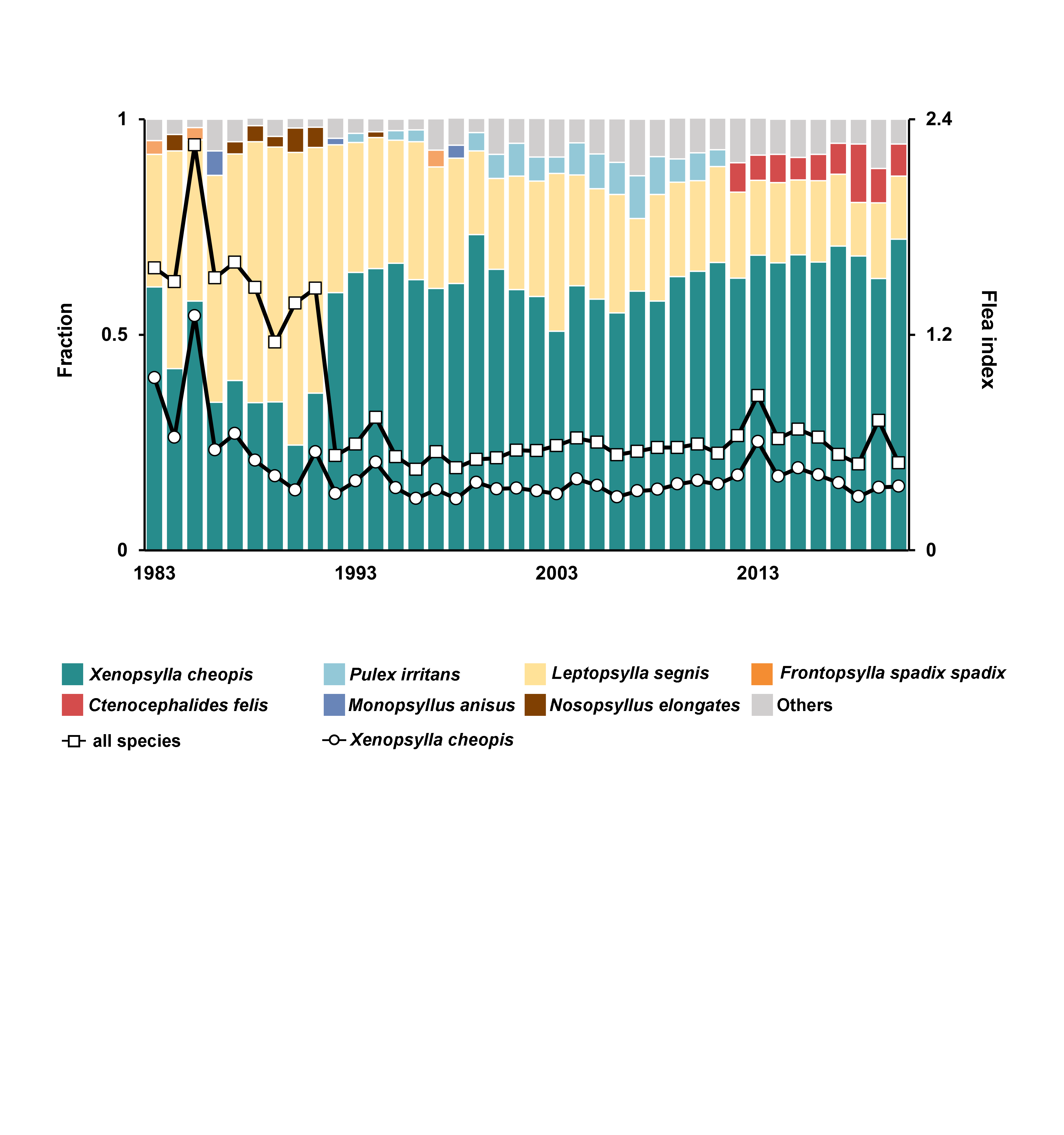

Supplement: S6 Fig — Same with Fig 2B but focus on the flea species isolated from the main host in domestic foci (Rattus tanezumi). (TIF) [file pntd.0011317.s007.tif]

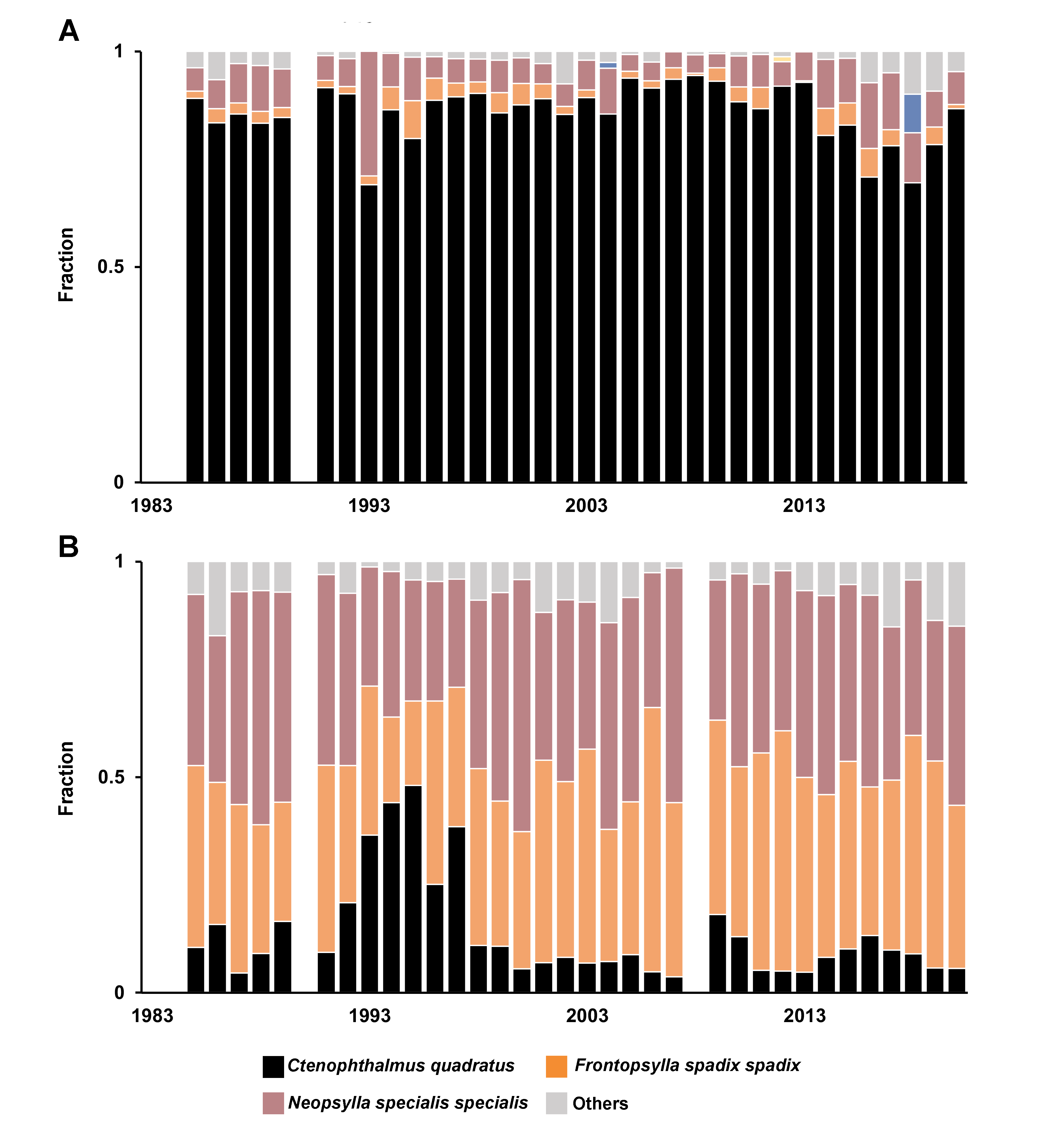

Supplement: S7 Fig — Same with Fig 2B but focus on the flea species isolated from main hosts in wild foci, i.e. (A) Eothenomys miletus and (B) Apodemus chevrieri. (TIF) [file pntd.0011317.s008.tif]

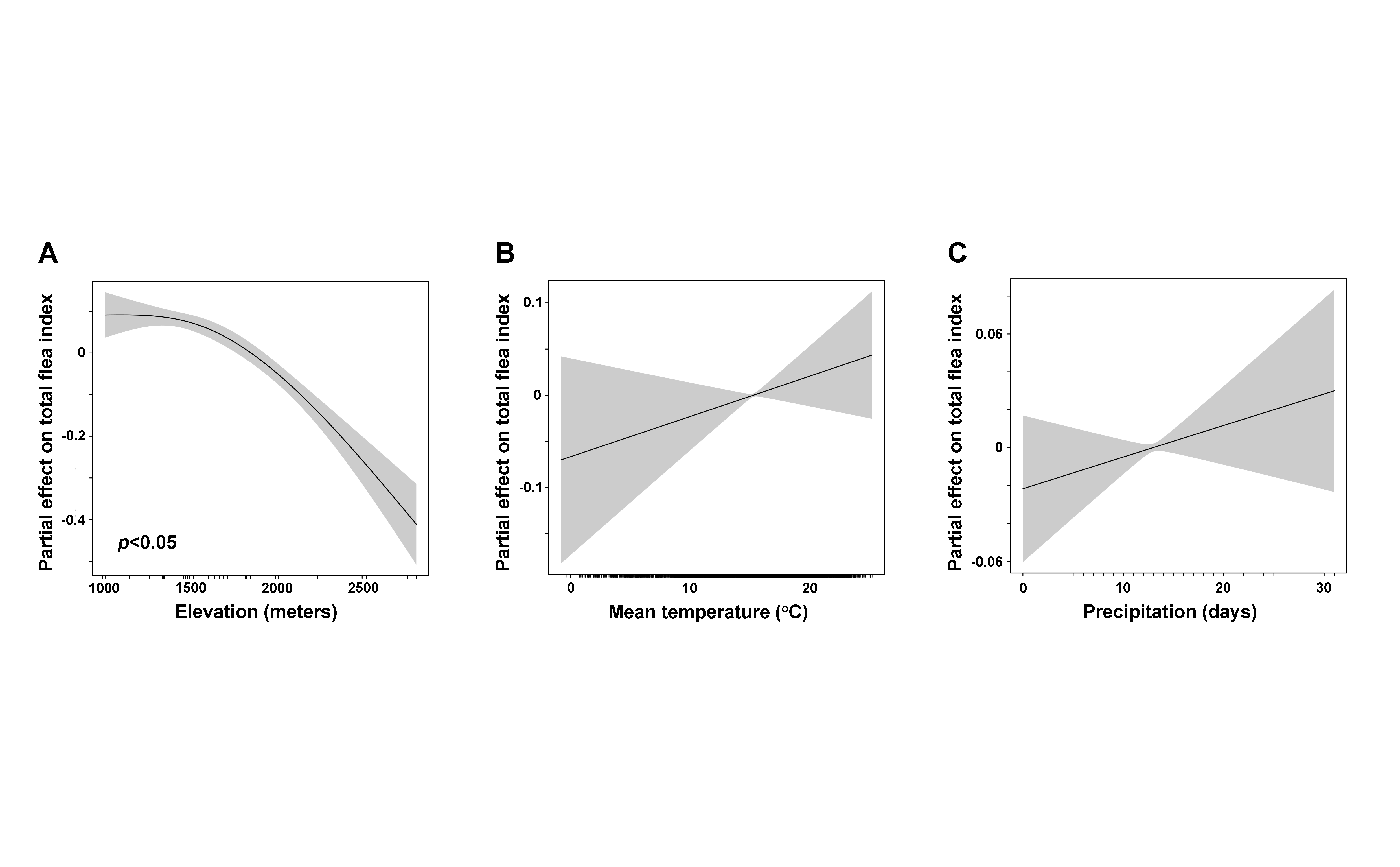

Supplement: S8 Fig — (A) The partial effects of elevation and meteorological conditions i.e. (B) monthly mean temperature and (C) precipitating days in the previous month on VFI are quantified using GAM. (TIF) [file pntd.0011317.s009.tif]

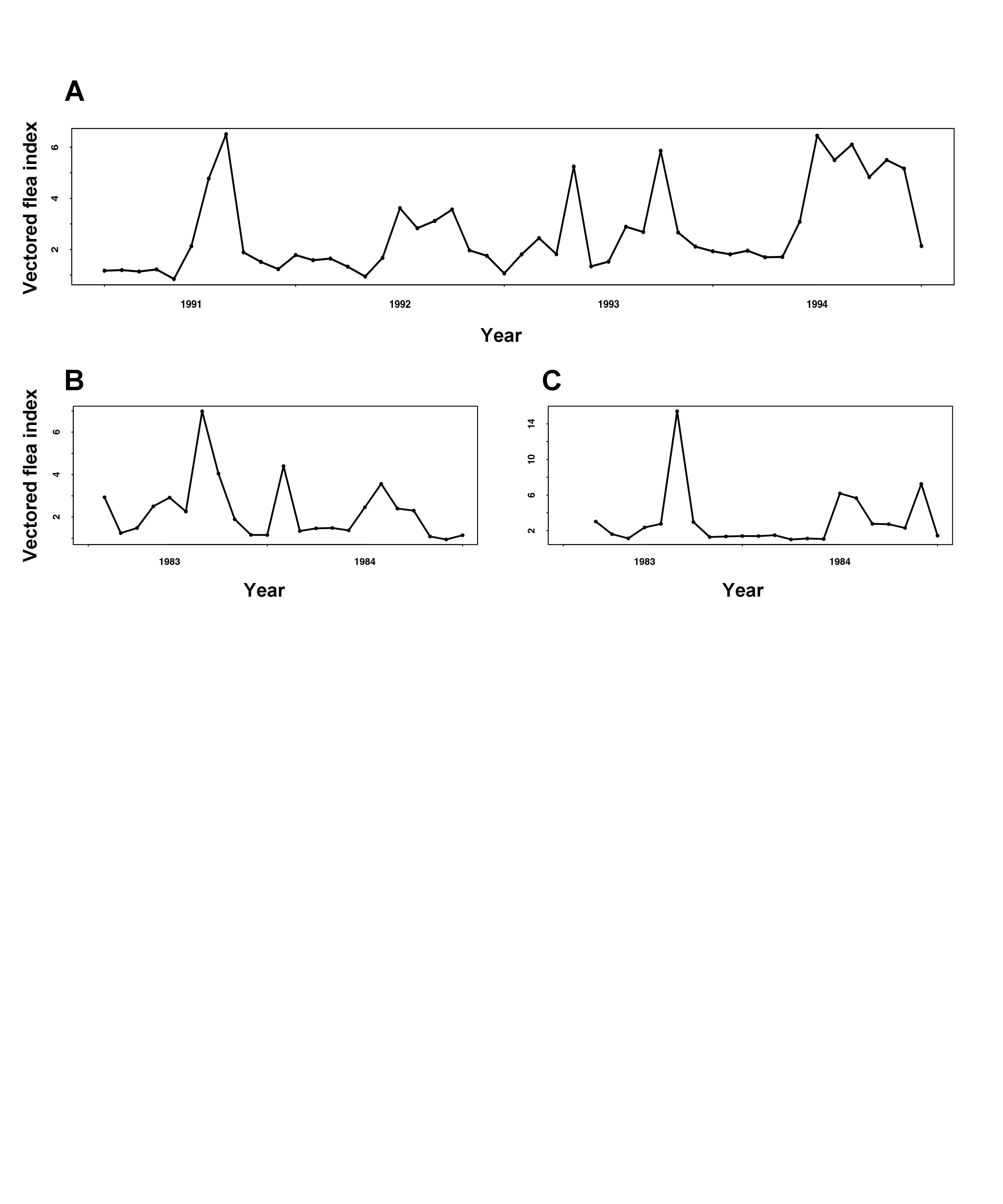

Supplement: S9 Fig — i.e. (A) Yingjiang, (B) Ruili and (C) Longchuan. (TIF) [file pntd.0011317.s010.tif]

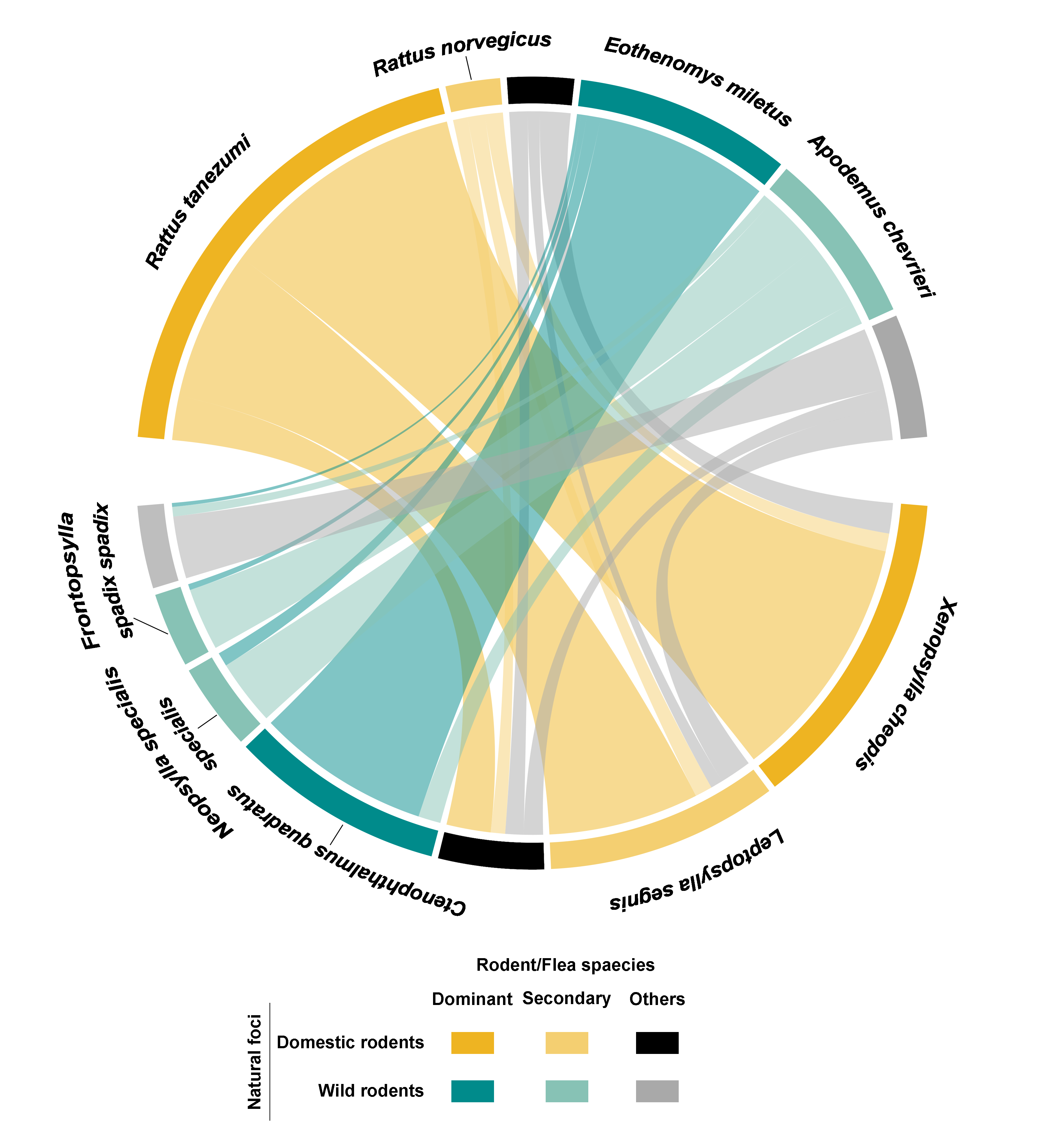

Supplement: S10 Fig — The dominant, secondary and other species of rodents and fleas in domestic (yellow) and wild (green) rodent foci are shown. The size of the arc of sectors is the fraction of species in the population. (TIF) [file pntd.0011317.s011.tif]

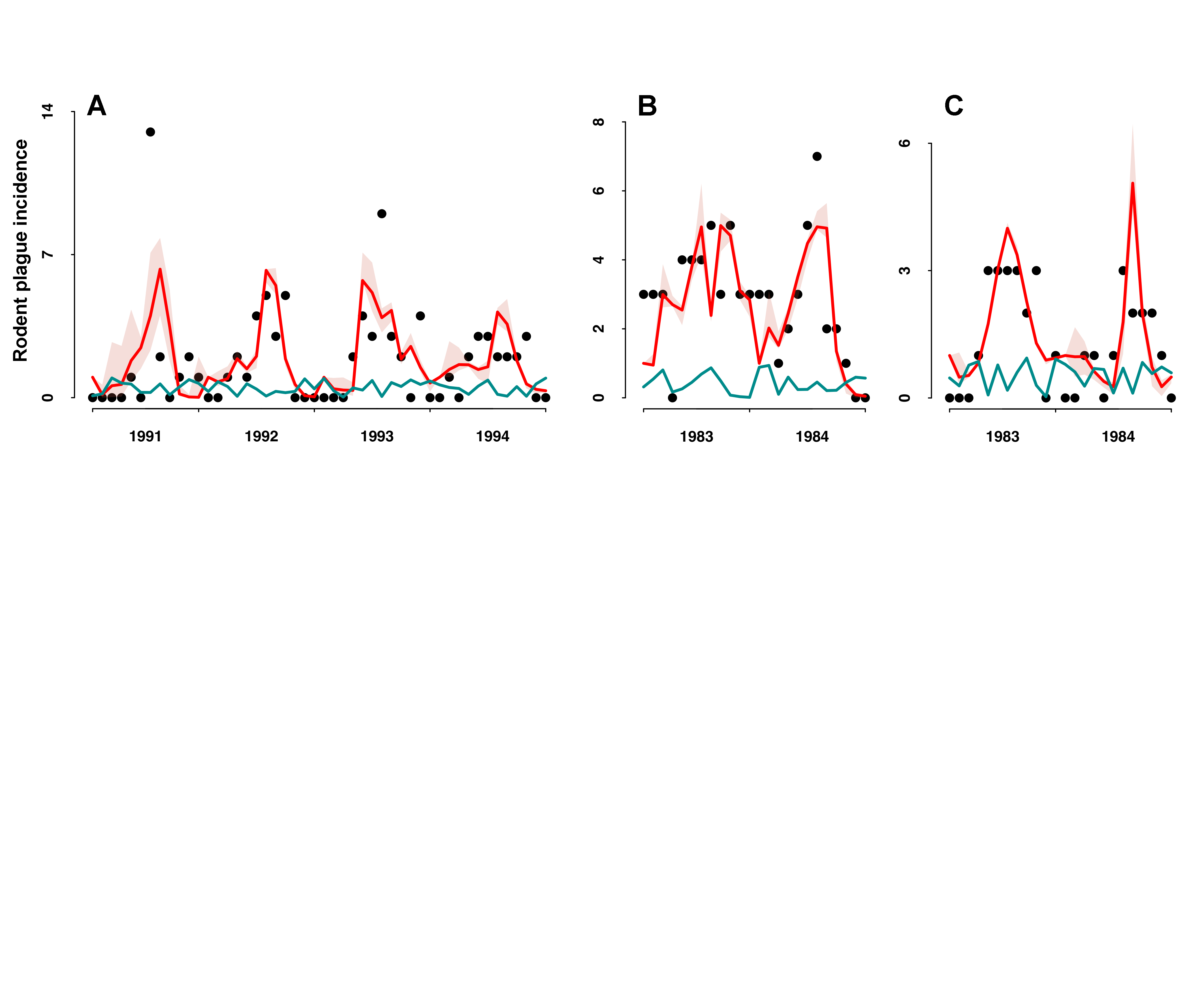

Supplement: S11 Fig — Estimates of rodent plague in (A) Yingjiang, (B) Ruili and (C) Longchuan by the main model and alternative model are presented by red and green curves, respectively. Observed number of rodent plague are shown by points. (TIF) [file pntd.0011317.s012.tif]
