# Supplementary material for: Associations between ecological diversity and rodent plague circulation in Yunnan Province, China, 1983–2020: A data-informed modelling study
Source: PLoS Negl Trop Dis. 2023 Jun 22;17(6):e0011317. doi: 10.1371/journal.pntd.0011317 (PMC10287002; doi:10.1371/journal.pntd.0011317)
Supplement: S3 Table — The performance of GAM in selected (i.e., the 1-month lag model) and all counties (i.e., the full model) in two foci is quantified by the generalized cross-validation criterion (GCV), proportion of deviation explained by model and R2. (DOCX) [file pntd.0011317.s015.docx]

**S3 Table. Statistical model performance in fitting VFI.** The performance of GAM in selected (i.e., the 1-month lag model) and all counties (i.e., the full model) in two foci is quantified by the generalized cross-validation criterion (GCV), proportion of deviation explained by model and R^2^.

| **Model** | **Model formular** | **GCV** | **Deviance explained (%)** | **R^2^** |
| --- | --- | --- | --- | --- |
| 1-month lag | $V_{t}=a_{t,i}+b(E_{i})+c(T_{t-1,i})+d(P_{t-1,i})+e(V_{t-1,i})+\varepsilon_{t,i}, i=1,..., 34$ | 8884·11 | 47 | 0·446 |
| Full model | $V_{t}=a_{t,i}+b(E_{i})+c(T_{t-1,i})+d(P_{t-1,i})+e(V_{t-1,i})+\varepsilon_{t,i}, i=1,..., 51$ | 9888·40 | 44·9 | 0·419 |
